# Neuroendocrine Influencers and Associated Factors That Shape Jaw Movement and Growth in Temporomandibular Joint Disorder Management: A Systematic Review of Clinical and Radiographic Evidence

**DOI:** 10.3390/jpm13050840

**Published:** 2023-05-16

**Authors:** Taseef Hasan Farook, James Dudley

**Affiliations:** Adelaide Dental School, Faculty of Health and Medical Sciences, The University of Adelaide, Adelaide, SA 5005, Australia

**Keywords:** jaw movement, arthritis, disc derangement, radiomics, biomarkers, hormones

## Abstract

Objective. To investigate the influence of endogenous and exogenous neuroendocrine analogues on the range and motion of jaw movement, mandibular growth, and factors affecting condylar guidance in patients with temporomandibular joint disorders using clinical assessment and radiographic imaging. Material and Methods. Eligible articles were extracted from eleven databases in early 2023 and screened following PRISMA protocols. Certainty of evidence and potential biases were assessed using the GRADE approach. Results. Nineteen articles were screened, with four deemed to be of high quality, eight of moderate quality, and the remaining seven of low to very low quality. Corticosteroids improve maximal incisal opening but not TMJ disorder symptoms. Higher doses worsen jaw movement and cause osseous deformity. Growth hormone affects occlusal development, and delayed treatment affects arch width. Sex hormone correlation with TMJ disorder is complex, with some studies showing a correlation between menstrual cycle phases and pain/limited mobility. Conclusions. The evaluation of neuroendocrine influencers in relation to jaw movement in patients with temporomandibular joint disorders involves the complex interplay of potentially confounding factors that each require careful consideration to ensure accurate diagnoses and evaluations.

## 1. Introduction

It has been demonstrated that interactions between biopsychological factors, soft-tissue function, and occlusion exert an influence on the symmetry and extent of jaw movement, which has conventionally been viewed as a purely mechanical process [1,2,3]. For example, in normo-occlusion, articular loads can reach near maximum values at half of the maximum muscle tension, while the force of occlusion and status of residual dentition affected the bone mineral density of the temporomandibular joint (TMJ) [4].

The TMJ is a secondary joint that caps after mandible formation, is unique to mammals, is absent in reptiles, and in mice lacks a true articular eminence [5]. The site and rate of growth are determined by genetics, endocrine influence, and are interdependent on daily jaw motion routines such as mastication and swallowing [6,7]. TMJ movement and occlusion in humans is therefore, unsurprisingly, very specific to endogenous and exogenous neuroendocrine phenotypes [8].

Disorders in the TMJ complex have a prevalence of 5–12% in the global population and exhibit a higher incidence in biological females, with a 25–40% increased susceptibility observed during TMJ development [9]. One in approximately every three patients will experience symptoms related to TMJ disorders, while disc displacement can be reported in one third of all asymptomatic individuals seeking dental treatment [4]. The management of conditions relating to the temporomandibular joint (TMJ) and occlusion poses a significant challenge, as experts remain divided over the longstanding debate surrounding the purported relationship between TMJ disorders and occlusion [10]. Although certain types of malocclusions, such as unilateral crossbite, have not been linked to disc derangement, it is evident that occlusal status and malocclusion have a noteworthy impact on condylar morphology, thus leading to an asymmetrical mechanical movement [11,12]. Degenerative and inflammatory disorders remodel the TMJ complex through destructive events such as condylar erosion, disc derangements, and joint effusions, and can serve to alter jaw motion [13,14,15]. Jaw movement behaviour is also affected by mandibular length, growth, and facial height parameters, which have demonstrated a history of exhibiting both positive and negative responses to therapies directed at neuroendocrine pathways [4,16,17,18]. The neuroendocrine system comprises endocrine organs that secrete hormones and similar substances once signalled by the neurological system, often serving as biomarkers in diagnosing TMJ pathologies [19,20].

Biomarkers are a discernible attribute and are quantitatively measured to indicate pathophysiological processes or responses to therapeutic interventions in an objective manner, and in the current context are related to the jaw movement process [5,9,21,22]. Moderate chewing forces promote neurological activity and cognition, whereas masticatory hyperfunction from parafunctional habits, trauma, and joint degeneration may lead to TMJ disorders by reducing acetylcholine activity and promoting anti-stress effects at the expense of joint space inflammation [9,23]. In such scenarios, the introduction of exogenous analogues is frequently performed to locally upregulate endogenous corticosteroids and other hormonal imbalances and trigger anti-inflammation [24]. Other neuroendocrine factors such as somatotrophins, sex hormones, gonadotrophins, thyroid analogues, parathormone, and its hormonal precursors also play a role in shaping the TMJ complex by disrupting proteoglycan synthesis, increasing the frictional mobility of the joint, causing condylar asymmetry, altering the physiologic growth of the TMJ complex, and predisposing patients to degenerative changes and derangement that eventually impair the mobility of the jaw [9,25,26,27,28].

Neuroendocrine molecular biomarkers, which rely on tissue, saliva, blood serum, or synovial fluid as a sampling source, are often coupled with adjunct diagnostic markers such as mi-RNA biomarkers or sensory marking [9]. In the TMJ clinics, such modalities are seldom used, and the application of adjunct clinical examination and medical imaging is preferred. This is because tumours and occlusal characteristics, such as trauma, lead to a hook effect and abnormal neuroendocrine readings [29]. Consequently, having radiomic data to support neuroendocrine profiling in the TMJ–occlusion complex is imperative, yet infrequently documented in published experiments. A systematic consolidation of the existing knowledge base on neuroendocrine profile manipulation, radiomic characterisation, and clinical jaw movement parameters is required but has not yet been completed. Hence, the present systematic review assessed the impact of neuroendocrine stimulation on clinical and imaging parameters that affect human jaw growth and movement, with a detailed discussion of the possible associated factors involved in management outcomes.

## 2. Material and Methods

### 2.1. Protocol and Registration

The study was reported according to the Preferred Reporting Items for a Systematic Review and Meta-analysis (PRISMA) protocol and subsequently registered with the International Prospective Register of Systematic Reviews (PROSPERO CRD 42023394568).

### 2.2. Eligibility Criteria

The following inclusion criteria were set for all articles:

- The article must contain empirical research documenting the manipulation of endogenous hormonal secretion or exogenous synthetic analogues on jaw movement or condylar morphology in a cohort consisting of at least five human subjects.
- The effects of neuroendocrine hormones historically responsible for shaping jaw movement or condylar morphology should be examined. These include pituitary secretions, growth hormones, catecholamine, gonadotrophin, sex hormones, corticosteroids, or other relevant hormonal precursors.
- The article should document computerised or tomographic imaging of the head-neck region. These reports should focus on factors associated with jaw movement parameters and report bilateral condylar profiling.
- Articles were excluded based on the following criteria:
- Research that reports only the localised influences of single tooth or single arch orthodontic morphometrics and skeletal relationships, manual methods of motion analysis using mechanical articulators or axiographs, pre-surgical procedures, or orthodontic disturbances to the TMJ and masticatory muscle system without a combined radiomic and neuroendocrine profiling component.
- Studies where patients and participants received antimicrobial or non-steroidal anti-inflammatory treatment as intervention therapy.
- Articles that report independent analyses of autoimmune inflammatory mediators, genetic or epigenetic variables, and subsequent syndromic manifestation, or neoplastic developments in the maxillomandibular region without a neuroendocrine component.

### 2.3. Study Characteristics

This study focused on the influence of neuroendocrine hormones on the temporomandibular joint (TMJ) and occlusion, specifically vertical and horizontal jaw movement, mandibular growth, and condylar guidance. The participants of this study were human subjects and the data generated were either retrospective or prospective. The collected data included neuroendocrine profiling and head-neck imaging, which were used to characterise TMJ and jaw movement. The exposure variable in the current review was the outcome that resulted either from exogenous neuroendocrine intervention or the monitoring of endogenous endocrine parameters on factors that influence jaw motion outcomes. The control group was established by collecting baseline values at day zero for the subjects scheduled to receive the intervention, as well as outcomes from alternate therapies or environmental alterations. Owing to the large heterogeneity within the included papers, a meta-analysis was not performed. Outcome measurements were direct influencers (such as maximum incisal or jaw opening, lateral excursions, protrusive action, mandibular growth profile, or condylar guidance) or indirect (such as analogue scales, scoring, or imaging signal intensity obtained from TMJ and condylar motion).

### 2.4. Information Source and Search Strategy

In February 2023, two reviewers retrieved data from various databases dating back indefinitely, using Boolean Logic [30] and wildcards [31]. The databases included Ovid Embase, Scopus, PubMed, Web of Science Core Collection, Current Contents Connect, Derwent Innovations Index, KCI-Korean Journal Database, Russian Science Citation Index, SciELO Citation Index, and EBSCOHost DOSS. A detailed account of all search methods applied has been documented in the Supplementary File, available online.

### 2.5. Data Extraction

The screening process followed PRISMA guidelines. Duplicates were removed and manuscripts were screened using a professional systematic review screening platform (Covidence.org; Veritas Health Innovations Ltd., Melbourne, Australia) that ensured complete agreement between the reviewers before allowing eligible manuscripts to advance through the screening process. Interactive troubleshooting and the translation of eligible foreign articles were performed in the presence of a deep learning language model (ChatGPT; OpenAI, San Francisco, CA, USA).

### 2.6. Risk of Bias Assessment

The Cochrane Grading of Recommendations Assessment, Development and Evaluation (GRADE) approach was used to assess the certainty of evidence and severity of potential biases using an online tool (GRADEpro GDT; Cochrane, UK).

## 3. Results

**Figure 1:**
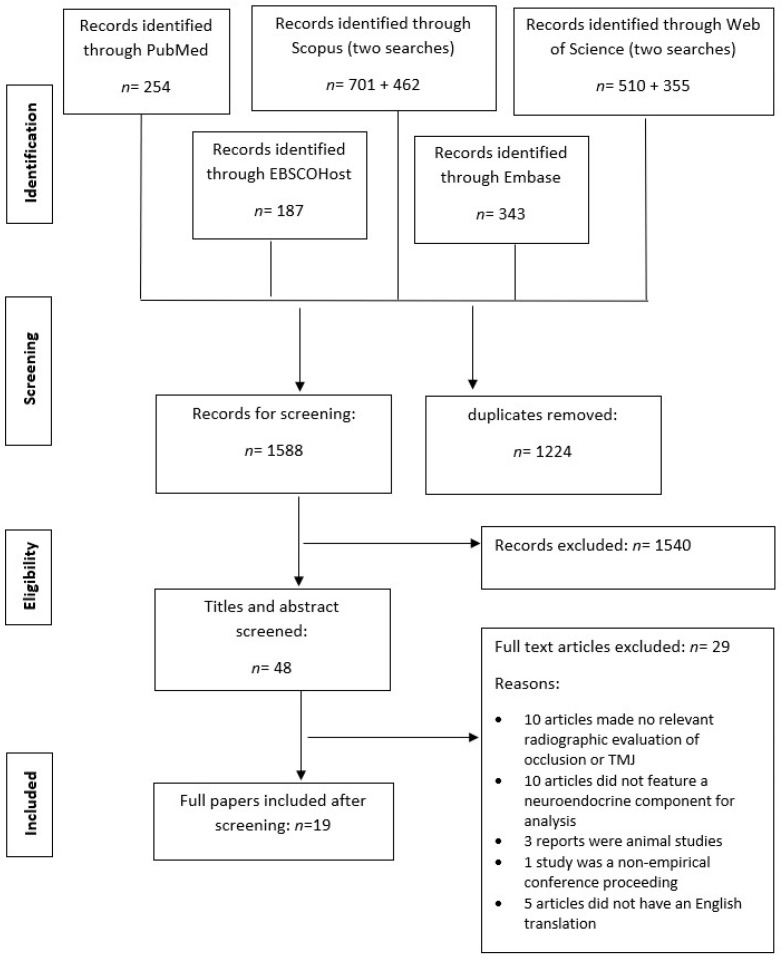
PRISMA flow diagram.

After screening (Figure 1), nineteen articles were included for full paper review. Following critical appraisal, four articles [32,33,34,35] were deemed to be of high quality, eight [36,37,38,39,40,41,42,43] of moderate quality, and the remaining seven to be of low to very low quality [44,45,46,47,48,49,50]. A detailed report of the appraisals is provided in the Supplementary File. The individual findings of all 19 articles have been presented in Table 1 and consolidated in the following paragraphs.

The effectiveness of corticosteroid injection on mouth opening was found to be significant (r = 0.52, *p* = 0.008) compared to lavage only, with no significant changes observed in dysfunction and symptoms [44]. Moderate abnormalities in the temporomandibular joint (TMJ) showed progression in symptomatic deterioration regardless of treatment with hyaluronate (35.3%) or betamethasone (36.8%) [33]. Patients under the age of 6 with degenerative changes responded best to exogenous corticosteroids with improved maximal incisal opening (*p* = 0.22). Follow-up studies on corticosteroid injection showed that 80% of patients did not demonstrate bony changes, but joint effusion was observed in 13 TMJs, with only 48% resolving on follow-up [45]. Exogenous corticosteroid administration was found to cause worsening of jaw movement in 27% of patients [38]. There was no significant difference (F = 0.94, *p* = 0.430) in lateral movement and maximum mouth opening between patients treated with corticosteroids and those treated with hyaluronate or Cox-2 inhibitors [39]. Bone deformity showed no improvement in 79% of patients, and only 31% showed improvement in maximal incisal opening > 5 mm after 2 years of repeated corticosteroid administration, with an average 2 mm maximum incisal opening improvement and no statistically significant inflammatory change after 2 years [36]. Higher doses of corticosteroids resulted in an increased deterioration (18%) of advanced lower jaw asymmetry after 7 months, with 41% of cases showing greater osseous deformity [32]. As a result, TMJ condyles that were completely damaged received significantly larger amounts of intra-articular corticosteroid (23 ± 9 mg) than other TMJs (6 ± 7 mg) [32]. Jaw deviation was reported in 20% of patients compared to a baseline of 40%, but jaw asymmetry saw an increase (16%) following corticosteroid application compared to baseline (12%) [37]. Ten of the fifteen patients showed progressively worsening bony changes following corticosteroid application, but with an increased maximum incisal opening of +3.5 mm [37].

A daily dose of the exogenous growth hormone analogue (pegvisomant) reduced IGF levels from 408 ± 114 to 199 ± 80 micro-g/L [34]. Delaying growth hormone treatment reduced its benefits to the occlusal development of the arch width in the posterior segment of both jaws after 5 years (−0.85 ± 0.62 to −1.21 ± 0.64). Maxillary anterior arch width development seemingly benefitted from delaying growth hormone treatment by one year (−1.07 ± 0.68) [40]. Females had significant negative correlation of IGF in mandibular growth (r = −0.64, *p* = 0.025) compared to males (+0.23, *p* = 0.34) during the descending phase of IGF (near the end of the growth spurt) [41].

TMJ disorders, particularly disc derangements, were related to sexual maturation but not to anxiety, depression, bruxism, and parafunctional habits [46]. No significant correlation of erosion to age or differences in gender existed when evaluating sex hormones. Testosterone levels were not significantly correlated to any inflammatory markers [35]. TMJ pain and limited mobility were rated higher in menstrual (55.0 ± 17.1) and secretory phases (60.0 ± 18.1) than in the proliferative phase (60.0 ± 18.1) [42].

IL-6 levels were significantly higher for TMJOA (13.80 ± 7.69) compared to DDwR (4.05 ± 3.13) and DDwoR (6.47 ± 5.85), but there was no significant correlation with leptin (r^2^ = 0.056, *p* > 0.05) in TMD patients [43]. Cholecalciferol among patient population (75.4%) was 23.6 ± 10.5 ng/mL and was below the baseline for deficiency [49]. The visual analogue scale for pain intensity during jaw opening and biting did not correlate significantly with the level of endorphins present. Endorphin levels were not significantly associated (*p* = 0.393) with the degree of bone remodelling without synovitis (0.18 ng/mg) and with severe synovitis (0.13 ng/mg) [50]. Pain intensity over masseter muscles correlated (r = −0.53, *p* = 0.028) significantly with jaw movement [47].

## 4. Discussion

The present review provides a comprehensive report on the various endocrine factors that impact the growth and movement of the temporomandibular joint (TMJ). Furthermore, the study discusses the associated factors that shape the endocrine influence on the TMJ. One strength of this study lies in its methodological approach to identifying both exogenous and endogenous hormones and their associated factors. This was accomplished using an automated, cloud-based, language bot-assisted screening process, which facilitated full interrater agreements and mitigated the effects of factors such as resource limitation, screening fatigue, language, and selection biases.

While publication bias could not be eliminated, the study sought to minimise its effects by creating database-specific logic grids, conducting repeated searches in select databases using alternate logic grids, and utilising rapid automated screening. However, the lack of uniformity in the reporting techniques employed in the analysed articles and the scarcity of high-quality studies constrained the study. Additionally, the heterogeneity across the evaluations of endogenous and exogenous analogues made it impossible to conduct a meta-analysis.

Nevertheless, the discussion below endeavours to elucidate the intricate relationship that each neuroendocrine factor has with the TMJ and jaw movement, while reflecting on the associated factors such as diagnostic biomarkers and pharmacotherapy that influence their effects.

### 4.1. The Associated Factors in TMJ Disorders

Effective diagnoses of TMJ disorders require the use of medical imaging and specialised tests, such as electromyography (EMG), alongside biomarker assays as pain reporting relies on patient-dependent subjective scales that are easily manipulated and not interchangeable [18]. In clinics, muscle disorders accompanied by arthralgia represent the highest prevalence of diagnoses at 35%. Additionally, TMJ disorders not classified under any Research Diagnostic Criteria (RDC) account for the highest prevalence of such disorders in the community, at 28% [4]. The cardinal features of acute inflammation, such as swelling, redness, and increased temperature, are not always obvious in cases of TMJ inflammation [12]. TMJ inflammation is sometimes correlated with rheumatoid arthritis, and diagnosing the latter in the jaw requires an assessment of biomarkers such as rheumatoid factor, ani-citrullinated protein antibody, C-reactive protein, and erythrocyte sedimentation rate throughout symptom duration [20]. Crepitus is the most common clinical symptom of TMJ osteoarthritis, and radiomic features include osteophytic lipping, cyst formation, condylar flattening, and decreased ramus height [51]. However, the true shape of the condyle may not be recognized solely from panoramic radiographs [20]. Technetium bone scans, although commonly used for TMJs, have high false-negative rates, and the limitations of different imaging modalities such as cone beam computed tomography (CBCT), multidetector computed tomography (MDCT), magnetic resonance imaging (MRI), ultrasound (USG), and positron emission tomography (PET) should be considered [20]. MRI scans are particularly useful for detecting inflammation-induced disease activity. However, clinical findings may not always be consistent with image interpretations, and clinicians find themselves relying upon qualitative scalar feedback from patients [36]. The included Supplementary File details the imaging modalities used in the studies included in the current review.

Asian populations have a higher prevalence of mandibular prognathism and class III occlusal relationships than Caucasian populations, with familial incidence being higher for mandibular prognathism than normal occlusion [16]. An interplay between genetic and environmental factors is responsible for facial height and mandibular morphology, reflected on cephalograms by Ar-Pog measurements, which may be responsible for jaw movement and translation. Epigenetic mechanisms such as DNA methylation, histone modification, and microRNA can cause abnormal joint remodelling and TMJ destruction [8]. A decreased expression of lubricin can distort the hourglass shape of the articular disc in the intermediate zone, [5] while *Col2* and *Adamts1* genes may be responsible for mandibular prognathism in Chinese populations, and masticatory muscle load can affect the growth of the mandible and maxilla [8,16]. Orthognathic surgery can also alter the mRNA compositions of masseter muscle, suggesting that a history of surgery is an important factor to consider when evaluating jaw movements [16].

Important neuroendocrine biomarkers reflecting gene inheritances, such as catecholamine, oestrogen, folate, and HLA, can lead to the diagnosis of degenerative disorders, and genetic polymorphism causing persistent TMJ pain and altered jaw motions [8]. Genetic alterations also increase proteoglycan secretion, leading to the secretion of matrix-degrading enzymes that degrade type 2 collagen in TMJ, while alterations in cholecalciferol uptake can lead to degenerative disorders, reduce bone thickness, and increase inflammatory mediators [8]. Excess bone remodelling and degradation of cartilage in TMJ result from transforming growth factor (TGF) increase, and osteophyte formation on TMJ can occur due to the overproduction of epithelial growth factor (EGF), often caused by the absence of inhibitor genes. Additionally, mutations in fibroblast growth factor (fgfr3) cause retarded growth, and decreased expression of lubricin can cause degenerative diseases [5]. Finally, the control of *Trps1* and *Ihh* by parathyroid hormone-related protein (PTHrP) represses *Runx2* and causes a delay in mandibular chondrogenesis and abnormal hypertrophic chondrocytes [5,8].

Determining the source of molecular biomarkers is essential for identifying TMJ disorders. These biomarkers can be detected in various tissues, saliva, blood serum, or synovial fluids. Inflammatory cytokines, interleukins (IL), tumour necrosis factors (TNF), proteinases, and bradykinin are some of the molecular biomarkers that can indicate inflammation and degenerative disorders [9]. Several types of biomarkers have been identified to report on chronic pain and inflammation in areas of the body other than TMJ. For instance, matrix metalloproteinases (MMP) can be detected in urine samples but are more closely correlated with pelvic pain syndrome in women. Contrastingly, salivary cortisol biomarkers have been shown to be useful in reporting chronic musculoskeletal pain by demonstrating lower levels of cortisol in the morning and evening. In addition, specific salivary biomarkers have been found to decrease in cases of temporomandibular joint (TMJ) disorders, including cortisol, alpha amylase, nerve growth factor (NGF), and brain-derived neurotrophic factor (BDNF). Salivary biomarkers have also been identified for dental and periodontal inflammation, such as an increase in IL-1, IL-6, and cortisol, and a decrease in CRP and alpha-2-macroglobulin. Burning mouth syndrome has its specific salivary biomarker indicators, including an increase in alpha amylase, IgA, and macrophage inflammatory protein-4 (MIP4), and a decrease in uric acid and ferric reducing activity of plasma (FRAP) [18]. Neurotransmitters such as histamine, serotonin, glutamate, and adenosine, and pain-inhibitory neurotransmitters such as calcitonin gene-related peptide, GABA, opioid peptides, and cannabinoids can also be detected in clinically painful, degenerative TMJs [9]. Interestingly, no correlations were documented between cytokine biomarker quantity and the severity of symptoms of TMJ disorders [51].

Insulin-like growth factor (IGF) and hormonal neuropeptides such as cortisol and endorphins can be found in increased quantities during periods of stress that can predispose to TMJ pain dysfunction [9]. IL-1 beta, IL-8, TNF, IL-6, *Cox-1.2*, PGE2, LTB4, VEGF, *MMP-1*, *MMP-2*, *MMP-3*, *MMP-8*, and *MMP-9* are biomarkers prominent in cases of internal derangement, while IL-10 and IL-6 are detected at higher baseline levels in the synovial fluid for successful outcomes and poorer outcomes in TMJ arthroscopy, respectively [12]. The development of rheumatoid arthritis is often attributed to the presence of histocompatibility antigens HLA-DR1 and HLA-DR4, while IL-1beta, rarely found in healthy synovial fluid, may be responsible for inflammatory reactions, pain, allodynia, and hyperalgesia in TMJ, and prevents the synthesis of proteoglycans [4]. Serum levels of C-reactive protein and IL-1beta are also elevated in patients with the condition [4]. Joint effusion is associated with growth factors (BDNF, FGF, IGFBP), stromelysin, and MMP-inhibitor, while joint movement pain and degradation are associated with TIMP-1, *ADAMT-4*, and *ADAMT-5* biomarkers [12]. Moreover, aggrecan proteoglycan is identified in the TMJ, and higher levels of aggrecan proteoglycan are found in patients with chronic closed lock disc derangement [12].

The treatment of TMJ disorders involves various medications and therapies. NSAIDs are effective for TMD but contraindicated in patients with gastrointestinal infections where *Cox-2* inhibitors are often prescribed [24,39]. Codeine and hydromorphone are useful for severe cases due to the existence of peripheral subtypes of opioid receptors in TMJ [24]. Muscle relaxants are not more effective than placebos for TMJ disorders but can surprisingly be combined with NSAIDs for promising outcomes [24,39]. Tricyclic antidepressants and selective serotonin reuptake inhibitors (SSRI) can reduce pain at doses lower than those used to treat depression, [24] but SSRIs can increase the tendency of bruxism and rates of dental implant failure [4,52]. Anticonvulsants such as gabapentin can reduce TMJ pain arising from muscles of mastication. Benzodiazepines enhance GABA response, induce muscle relaxation, and reduce stress-dependent corticosteroid release, but are generally discouraged, accounting for drowsiness and withdrawal symptoms [4,53]. Infliximab, a monoclonal antibody, can reduce IL-6 in post-treatment synovial fluid [4]. However, for autoimmune inflammatory disorders such as juvenile idiopathic arthritis (JIA), avoiding permanent mandibular and craniofacial growth disturbances remains the primary aim of successful treatment [21].

### 4.2. Corticotrophins and Corticosteroid

The management of temporomandibular joint (TMJ) disorders by locally regulating the corticosteroid pathway yielded varying results across different studies and for patients in different age groups. TMJ arthritis can clinically demonstrate a symptomatic limitation of incisal opening, lateral, or protrusive jaw movement with signs of inflammation in an imaging modality [36]. The intra-articular TMJ growth site responsible for vertical growth is unique to the joint and widely believed to be susceptible to growth suppression by local administration of exogenous corticosteroids [21]. For JIA patients across the 4 to 17 year age groups, there was no significant association between maximum incisal opening or jaw movement with other clinical variables [45]. The frequent need for the repeated administration of intra-articular triamcinolone was seen to achieve desirable jaw opening outcomes [32], sometimes with detrimental effects leading to worsened jaw movements and often requiring adjunct therapies with methotrexate and non-steroidal anti-inflammatory drugs (NSAID) [21,37,38]. For JIA patients with TMJ symptoms, it may take 11 months for patients to demonstrate clinical symptoms, from disease diagnosis to the onset of TMJ symptoms [37]. The usefulness of intra-articular endogenous corticosteroids for mandibular growth and the incisal opening parameters of jaw movement can be controversial as follow-ups are often at relatively short time intervals.

In adult patients suffering from TMJ osteoarthritis and disc derangement, sodium hyaluronate and *Cox-2* inhibitor injections provided an alternate albeit ineffective form of management as there were no significant differences compared with corticosteroid injections in terms of TMJ disease progression or resolution [33,39]. Combining corticosteroids with the local anaesthetic lidocaine for TMJ pain management temporarily resolved symptoms for 4–6 weeks. On the other hand, disease-modifying antirheumatic drug therapy may not play an active role in increasing maximum incisal opening [36]. In such instances, prescribing NSAIDs with corticosteroids can reduce gastrointestinal effects and extend anti-inflammatory efficacy [24,45]. There is still a high chance of asymptomatic joint effusion following the exogenous application of corticosteroids. However, this treatment provides minimal benefits to mandibular growth, jaw symmetry, and the long-term prognosis of retained jaw opening. Therefore, it is recommended that clinical follow-ups should be scheduled at least one year apart for the proper monitoring of any changes [21].

### 4.3. Somatotrophins

When evaluating growth hormone levels in adults, it was recommended to apply biomarkers across multiple stimuli due to the hormone’s undetectable levels between secretory spikes in healthy adults [29]. Additionally, the assessment of insulin-like growth factor 1 (IGF-1) is recommended to test for acromegaly, and plasma growth hormone releasing hormone (GHRH) testing is advised to rule out neuroendocrine tumours, while keeping in mind that these tests can be adversely affected by the oral intake of oestrogen supplements [29]. Conditions such as diabetes mellitus and liver cirrhosis may alter IGF-1 values by increasing the proteolysis of the IGF-1 binding protein 3 (IGFBP-3) [29].

Obstructive sleep apnoea (OSA) affects 1 billion people worldwide and is associated with multiple factors such as obesity, smoking, hypertension, and diabetes. OSA has been consistently associated with cervical spine pathologies and postural changes, predominantly anterior head extension along the cervical spine [22]. Patients with OSA also report comorbid TMJ disorders which may lead to decreased pain sensitivity. Furthermore, there is a strong correlation between OSA and temporomandibular joint osteoarthritis, which triggers similar inflammatory pathways of TNF-α and IL-6 [22]. One study that investigated the interplay between IGF-1, abnormal growth hormone levels, altered mandibular growth and form, and OSA, reported that OSA was present across 83% of patients with acromegaly [34]. A five-year study was conducted on prepubertal patients with normal growth hormone levels but stunted physical growth. Nutritional deficiency or psychosocial dwarfism were methodically excluded. The study found that annual changes in jaw growth and growth hormone levels were not statistically significant in most patients after exogenous administration of the growth hormone analogue [40]. However, immediate commencement of therapy was more beneficial than delaying treatment by one year [40]. It is crucial to recognise certain limitations, as although IGF-1 levels were reliably dependent on growth hormone prior to puberty, changes in sex hormone levels such as testosterone upon entering puberty could affect IGF-1 levels, leading to a cascade of additional differential diagnoses [41].

### 4.4. Gonadotrophins and Sex Hormones

The pituitary gland secretes gonadotrophins which stimulate gonads and sex hormone production. The primary gonadotrophins are follicle stimulating hormone (FSH) and luteinizing hormone (LH). These hormones have a role in spermatogenesis in males and ovarian development across the menstrual cycle in females. In the diagnosis of TMJ disorders in females with potential menstrual associations, fat-suppressed contrast-enhanced T1 sequence imaging has demonstrated superior efficacy in detecting posterior disc attachments. Additionally, endometrial dating, which categorises menstrual phases as menstrual (day 1–5), proliferative (day 6–14), secretory (day 15–26), and premenstrual (day 27–28), has been clinically utilised for the purpose of menstruation cycle evaluation [42].

The primary sex hormones that are noteworthy in TMJ diagnostics are oestrogen and testosterone. Physiological oestrogen is present in three forms, namely oestradiol, oestrone, and oestriol. While bioavailable oestradiol in men decreases over time, bioavailable oestradiol in women fluctuates throughout the menstrual cycle [26]. Hajati et al. [35] observed that median oestradiol in males above 60 years of age increased to 65 pmol/L, while in females it decreased to 41 pmol/L. Oestrogen plays a significant role in causing several oral health issues such as TMJ degeneration, gingivitis, periodontal diseases, and TMJ disorders. Although past research is inconsistent, more recent studies suggest that reduced oestrogen levels may contribute to the development of TMJ disorders [26]. The relationship between sex hormones and mental health can trigger a cascade of events culminating in the development of various psychological and somatic conditions such as generalised anxiety, depression, parafunctional bruxing, and somatic symptoms [46]. This in turn can exacerbate stress levels leading to a heightened secretion of corticosteroids, which can intensify the inflammatory response and contribute to the manifestation of symptoms associated with TMJ disorders.

The degeneration of mandibular condylar fibrocartilage cellularity occurs progressively and plateaus at around 50–60 years. Reduced endogenous oestrogen levels in post-menopausal women may be associated with such degenerative TMJ disorders [27], although advanced age and oestrogen levels did not appear to be as significant as sexual maturation in contributing to the likelihood of a patient developing disc displacement with or without reduction [28,46]. Normal levels of oestradiol in pre-menopause were found to be 100 to 1500 pmol/L but reduced to below 50 pmol/L in post-menopausal women. Testosterone, which is considered beneficial in the anti-inflammatory functions of TMJ and the occlusal complex, saw slight increases in women from 0.3 to 2.6 nmol/L, while it increased fivefold in men from 6 to 30 nmol/L with progressing age [35].

Previous reports involving animal models suggest that treatment with 10 nM of oestradiol may have a negative impact on joint lubrication, indicating that very high levels can reduce proteoglycan synthesis, increase the frictional mobility of the joint, and contribute to temporomandibular joint osteoarthritis [26]. Human analyses have suggested that hormonal variations can lead to symptomatic TMDs with condylar asymmetry as a potential cause [25]. Females were generally found to be more prone to right-sided condylar hyperplasia which leads to shunted mandibular growth [25]. Additionally, one study found that 86% of symptomatic TMD cases demonstrated signs of degenerative disorders [48]. Furthermore, individuals over the age of 65 with symptomatic TMD may have degenerative forms of the disease, with 45–70% exhibiting radiographic changes [27]. Yet, hormone replacement therapy for TMD is a relatively new area of research with limited studies available.

Finally, plasma glutamate is a biomarker that can indicate the severity and duration of inflammation in the body. Interestingly, plasma glutamate was reported to increase in males (0.5 to 7.1) and decrease in females (4.4 to 2.8) after 7 months following the radiographic diagnosis of TMJ arthritis [35]. This suggests that although oestrogen levels decrease in older females, the proportion of symptomatic cases may also decrease compared to males. It also suggests that males may experience symptomatic jaw movements and reduced voluntary openings at a later stage, while females may experience reduced involuntary jaw opening with low-grade inflammatory symptoms.

### 4.5. Hormonal Precursors

A deficiency of cholecalciferol is characterised by concentrations below 30 ng/mL or 75 nmol/L, which is a global issue with 42.1% of patients presenting with skeletal defects and 46.5% having some form of dentoalveolar malocclusion [49]. However, evaluating cholecalciferol is complicated by unavoidable heliophysics and geographic variables. Neuropeptides and calcitonin gene-related peptide (CGRP) have been found to correlate with pain intensity and surface lesions [51]. Critical care patients who are infused with ionic calcium during arterial blood gas evaluation are at risk of hypocalcaemia, which affects intact parathyroid hormone (iPTH) tests [29]. Furthermore, renal failure, altered glomerular filtration rate, and cholecalciferol deficiency can also affect iPTH tests. Therefore, serum calcium levels must be elevated simultaneously with parathormone levels. The renin–angiotensin–aldosterone system often affects the regulation of such precursors, with renin increasing in patients with hyperkalaemia, and mineralocorticoid receptor antagonist (MRA) antihypertensives and spironolactone needing to be paused before testing for renin function. However, calcium channel blockers and alpha blockers do not affect such tests [29,51].

### 4.6. The Limitations of Neuroendocrine Biomarkers in TMJ Diagnostics

Neuroendocrine biomarkers are capable of generating false normal values due to the type of assay used and several interplaying covariant factors, such as stress-induced variations and overlooked hormonal co-activities [29,47]. Therefore, appropriate modifications of biospecimens are necessary for accurate evaluations [29,50]. Early morning specimen collections are recommended for most hormones affected by the corticotrophin pathway, but the dysregulation of pain inhibitory pathways and hypothyroidism should also be considered [47]. A false elevation of pituitary and hypothalamic hormones can occur due to various factors, such as lactotrophic tumours, SSRI therapy, biotin interference with hormonal assays, and the cross-reactivity of steroidal hormones with immunoassays [29,50]. Diagnostic markers such as corticotrophin axis dynamic serum tests and HbA1c are limited by false readings during conditions such as obesity, stress, alcoholism, anorexia, elevated cortisol binding globulin levels, oestrogen, pregnancy, hyperthyroidism, chronic renal failure, and hypometabolism due to liver failure haemolysis, chronic lymphocytic leukaemia, nitrates and benzene derivative drugs, and vitamin C overload [29].

Testosterone deficiency can lead to dental symptoms including osteoporosis, decreased alveolar bone support, periodontal diseases, xerostomia and subsequent caries, and methods to avoid false readings include testing early morning total testosterone, sexual hormone binding globulin (SHBG), luteinizing hormone (LH), and follicle-stimulating hormone (FSH) [29,48]. To avoid false readings, SHBG readings should be interpreted with caution, as it can increase in hyperthyroidism and liver disease and decrease in hypothyroidism and obesity [29,48]. Adipose tissue hormones such as leptin are independent of the lactotrophic and corticotropic axes but similar to corticosteroids and can influence obesity by regulating appetite [43]. They are also responsible for energy expenditure alongside initiating TMJ inflammation and affecting the degree of jaw motion [43]. Daytime sleepiness can be linked to various factors such as cholecalciferol, thyroid, parathyroid, sex hormones, or relevant neuroendocrine precursors [46]. Yet, the assessment of the quality of one’s sleep necessitates an elaborate configuration of a polysomnography apparatus with measurements taken between specific hours and across a comprehensive range of modalities [34]. The clinical diagnosis of symptomatic TMDs requires a descriptive history and the consideration of psychosocial and behavioural factors [9]. Sleep quality evaluations are often absent in the diagnosis of TMJ disorders, thus limiting the certainty of evidence. The studies included in the present review overlooked the majority of the variables mentioned, highlighting the lack of a comprehensive tool that could assist dental experts in methodically documenting all the neuroendocrine factors linked to TMJ and occlusion for specific cases. This inadequacy underscores the need for further research to develop such a tool. Overall, the evaluation of neuroendocrine hormones in relation to jaw movement in patients with temporomandibular joint disorders involves the complex interplay of potentially confounding factors that each require careful consideration to ensure accurate diagnoses and evaluations.

## 5. Conclusions

The following key conclusions can be drawn from the findings of the current review:

1. Corticosteroid injection was found to be effective in improving mouth opening, but moderate abnormalities in the TMJ showed progression in symptomatic deterioration regardless of treatment with hyaluronate or betamethasone.
2. Exogenous corticosteroid administration was found to cause a worsening of jaw movement in more than a quarter of the patients studied, and higher doses resulted in increased deterioration of advanced lower jaw asymmetry.
3. Delaying growth hormone treatment reduces benefits to the occlusal development of the arch width in the posterior segment of both jaws after 5 years, but maxillary anterior arch width development seemingly benefits from delaying growth hormone treatment by 1 year.
4. TMJ disorders, particularly disc derangements, are related to sexual maturation but not to anxiety, depression, bruxism, or parafunctional habits.

## Figures and Tables

**Table 1:** Summary of findings.

Author, Year	Patient Characteristics	Source of Neuroendocrine Factor	Baseline Values of TMJ-Occlusion Complex	Outcome Changes in TMJ-Occlusion Complex
*Corticotrophins and corticosteroid*				
Antonarakis, 2018 [44]	41 Patients (36 females, 5 males) aged 13.6 ± 4.0 years and diagnosed with JIA	Exogenous intra-articular injection: triamcinolone acetonide 20 mg/mL alongside lavage in 21 patients. control (*n* = 8) was established with patients who underwent lavage only with 0.9% NaCl.	Corticosteroid + lavage (*n* = 41) = 35.7 ± 8.5 mm Lavage only (*n* = 8) = 37.4 ± 7.9	Corticosteroid + lavage (*n* = 41) = +2.3 ± 7.0 mm Lavage only (*n* = 8) = +1.4 ± 6.2 mm
Arabshahi, 2005 [45]	23 patients (20 females, 3 males) aged 4–16 years and diagnosed with JIA	Exogenous intra-articular injection: 16 patients were administered triamcinolone acetonide 40 mg. 7 patients administered triamcinolone hexacetonide 20 mg/mL.	MIO = 35.9 ± 7.25 mm	MIO = 40.7 ± 6.0 mm
Frid, 2020 [36]	15 patients (12 females, 3 males) aged 11–16 years and diagnosed with JIA	Exogenous intra-articular triamcinolone acetonide 20 mg/mL administered at 1–3 months, 1 year, and 2 years.	MIO = 44 mm	MIO change (2 months) = + 1 mm (45 mm)
Lochbuhler, 2015 [32]	33 patients (23 female, 10 males) aged 5.3 ± 1.9 years diagnosed with JIA	Exogenous intra-articular triamcinolone acetonide 6 to 20 mg/mL administered at 2–4 months and 6 to 12 months	Mandibular growth rate = 1.4 ± 0.1 mm/yr	Mandibular growth rate after 7 months = 0.7 ± 0.8 mm/yr
Moystad, 2008 [33]	40 patients (31 females, 7 males) aged 48.3 ± 13.5 years and diagnosed with TMJOA	Exogenous intra-articular betamethasone in 19 patients. Repeats were administered after 2 weeks	Treated TMJ scoring = 8.1 ± 8.7 Contralateral TMJ scoring = 5.9 ± 7.2	Treated TMJ scoring = 8.0 ± 6.8 Contralateral TMJ scoring = 5.1 ± 5.8
Ringold, 2008 [37]	25 patients (21 females, 4 males) of mean age 8.9 years diagnosed with JIA	Exogenous intra-articular injection: Triamcinolone acetonide 40 mg; Triamcinolone hexacetonide 20 mg/mL. The 25 patients received 74 injections on 47 occasions	MIO = 31.5 mm avg (ranging from 15 to 45 mm)	MIO change after 33.6 months = +3.8 mm avg (ranging from −18 to +20 mm)
Stoll, 2012 [38]	63 patients (43 female, 20 males) aged 8.5 ± 4.2 and diagnosed with JIA	Exogenous intra-articular administration of triamcinolone hexacetonide 20 mg/mL	MIO = 40.8 ± 0.93 mm	MIO change after 23 months = +2.7 mm avg (43.5 ± 0.90 mm)
Yavuz, 2018 [39]	44 patients (38 female, 6 males) diagnosed with disc derangement	Exogenous intra-articular administration of methylprednisolone acetate	MIO = 27.36 ± 4.57 mm	MIO change after 6 months = +6.3 mm avg (33.68 ± 4.81)
*Somatotrophins*				
Masoud, 2012 [41] ^a^	25 patients (12 female, 13 males) aged 9 to 18 years	Evaluation of endogenous GH and androgen activity by measuring IGF-1	Mandibular growth = 2.6 ± 1.35 mm	Mandibular growth after 5 years: When IGF-1 ≥ 250 μg/L, mandibular growth = 5.6 ± 2.88. When IGF-1 < 250 μg/L, mandibular growth = 2.13 ± 2.17.
Berg, 2009 [34] ^b^	12 patients (6 female, 6 males) aged 57 ± 15 and diagnosed with active acromegaly	Exogenous administration of 10–30 mg growth hormone antagonist, pegvisomant. Endogenous evaluation of IGF-1 serum assay	BMI-adjusted tongue volume = 105 ± 33 mL	BMI-adjusted tongue volume after 6 months = 83 ± 20 mL Mandibular length correlation to tongue volume = 90 ± 10 mm (r = 0.78, *p* = 0.003)
Richey, 1995 [40] ^c^	28 patients (6 females, 22 males) aged 5–9 years and diagnosed with idiopathic short stature independent of GH levels	Exogenous weekly administration of 0.1 to 0.3 mg/kg recombinant growth hormone Control (*n* = 28) was established with matched healthy individuals	Arch width Z score: Case = −1.51 ± 1.21 to −2.22 ± 1.03. Control = −0.75 ± 0.76 to −1.31 ± 1.39.	Arch width Z score after 5 years: Case = −0.19 ± 0.85 to −1.88 ± 0.27. Control = −1.00 ± 0.97 to −1.88 ± 0.27.
*Gonadotrophins and sex hormone*				
Shen, 2022 [46]	78 patients (43 female, 35 males) aged 13–14 diagnosed with anterior disc displacement (ADD)	Evaluation of endogenous oestradiol, progesterone, testosterone, FSH, LH, and prolactin	Healthy control (*n* = 362) MIO = 46.44 ± 5.87 mm MAP = 7.77 ± 2.22 mm MLE left = 8.66 ± 2.43 mm MLE right = 8.49 ± 2.08 mm FSH = 3.79 IU/L LH = 2.13 IU/L Oestradiol = 22.00 pg/mL Testosterone = 0.73 ng/dL Progesterone = 0.10 ng/mL Prolactin = 10.91 ng/mL	ADD patients (*n* = 78) MIO = 47.28 ± 6.04 mm MAP = 8.25 ± 2.27 mm MLE left = 8.75 ± 2.56 mm MLE right = 8.67 ± 2.08 mm FSH = 3.98 IU/L LH = 2.64 (IU/L) Oestradiol = 30.00 pg/mL Testosterone = 0.10 ng/mL Progesterone = 0.10 ng/mL Prolactin = 10.91 ng/mL
Gus, 2015 [48]	30 patients (all females) aged 27 to 34 years diagnosed with TMD	Evaluation of endogenous testosterone	Asymptomatic TMD profile Free androgen index = 1.6 ± 0.3 SHBG = 73.0 ± 35.9 Restricted jaw opening = 0% patients Deviations in mouth opening = 72% patients	Symptomatic TMD profile Free androgen index = 5.75 ± 2.1 SHBG = 45.2 ± 4.9 Restricted jaw opening = 36.4% Deviation in mouth opening = 72.7%
Hajati, 2009 [35]	47 patients (29 female, 18 males) aged 62 md, diagnosed with early RA	Evaluation of endogenous oestradiol and testosterone	-	Oestradiol (<50 pmol/L) and testosterone (<1.2 pmol/L) significantly correlated with CRP (<3 mg/L), glutamate, and TMJ erosions.
Suenaga, 2001 [42]	42 patients (all female) aged 14 to 44 years with symptoms of TMJ pain	Endogenous evaluation of the different phases of the menstrual cycle	Asymptomatic imaging signal intensity Menstrual phase = 0.55 ± 0.33 Proliferative phase = 0.87 ± 0.49 Secretory phase = 0.95 ± 0.33	Symptomatic signal intensity Menstrual phase = 1.74 ± 0.45 Proliferative phase = 1.16 ± 0.61 Secretory phase = 1.97 ± 0.63
*Other hormones and precursors*				
Leszczyszyn, 2021 [49]	114 patients (61 female, 53 male) aged 18 to 50 years	Evaluation of endogenous cholecalciferol (D3)	Endogenous levels without supplement = 20.25 ± 7.47 ng/mL Skeletal malocclusion without cholecalciferol deficiency (*n* = 28) = 21.4% of patients	Endogenous levels following supplementation with cholecalciferol 500–2000 IU/day supplements = 33.36 ± 11.89 ng/mL Skeletal malocclusion with cholecalciferol deficiency (<30 ng/mL) = 32.6% of patients (OR = 1.71)
Xiong, 2019 [43]	38 patients (31 female, 7 male) aged 17 to 63 years diagnosed with disc displacement (DD) or TMJOA	Evaluation of endogenous secretion of leptin within TMJ synovial space Control was established with healthy individuals (*n* = 7)	Findings from control (*n* = 7) MIO = 42.14 ± 4.95 mm Leptin = 157.41 ± 70.1 pg/mL	Outcomes for DDwR (*n* = 12) MIO = 39.75 ± 6.12 mm Leptin = 201.93 ± 90.73 pg/mL Outcomes for DDwoR (*n* = 13) MIO = 28.38 ± 6.83 mm Leptin = 227.04 ± 109.78 pg/mL Outcomes for TMJOA (*n* = 13) MIO = 32.46 ± 8.85 mm Leptin = 360.15 ± 157.28 pg/mL
Feldreich, 2012 [47]	18 patients (all female) aged 19 to 72 years diagnosed with chronic closed lock and were scheduled for joint discectomy	Evaluation of endogenous β-endorphin Healthy matched controls (*n* = 18) to establish baseline	healthy subjects (*n* = 18) β-Endorphin = 12.0 ± 0.67 pg/mL	Patients with symptomatic closed lock β-Endorphin = 17 ± 1.2 pg/mL MIO = 31.11 ± 8.86 mm
Kajii, 2005 [50]	38 patients (35 female, 3 males aged 16 to 78 years diagnosed with closed lock	Evaluation of endogenous β-endorphin	healthy subjects (*n* = 19) β-endorphin = 0.105 ± 0.055 ng/mg	Patients with symptomatic closed lock β-endorphin = 0.16 ± 0.08 ng/mg VAS score jaw opening = 56.84 ± 25.35

OR, odds ratio; md, median; MIO, maximum incisal opening; MAP, maximum anterior protrusion; MLE, maximum lateral excursion; JIA, juvenile idiopathic arthritis; TMJOA, temporomandibular joint osteoarthritis; RA, rheumatoid arthritis; TMD, temporomandibular disorder; IGF, insulin-like growth factor; GH, growth hormone; FSH, follicle stimulating hormone; LH, luteinising hormone; DD, disc displacement; ADD, anterior disc displacement; wR, with reduction; woR, without reduction; SHBG, sex hormone binding globulin; CRP, C-reactive protein. Maximum incisal opening (MIO) = interincisal opening + overbite. ^a^ Mandibular length (in mm) measured from condylion to gnathion on lateral cephalogram. ^b^ Mandibular length (in mm) measured from Tgo.h (horizontal distance from TMJ to gonion) to gnathion on lateral cephalogram. ^c^ Z scores measured for the number of standard deviations of reference sample. A score of 0 = patients were at expected stature given their age, −1.00 meaning patients were 1 standard deviation below normal.

## Data Availability

All data has been made available through the Supplementary Materials available with the online version of the manuscript.

## Supplementary Materials

The following supporting information can be downloaded at: https://www.mdpi.com/article/10.3390/jpm13050840/s1.
