# Supplementary material for: Neuroendocrine Influencers and Associated Factors That Shape Jaw Movement and Growth in Temporomandibular Joint Disorder Management: A Systematic Review of Clinical and Radiographic Evidence
Source: J Pers Med. 2023 May 16;13(5):840. doi: 10.3390/jpm13050840 (PMC10221279; doi:10.3390/jpm13050840)
Supplement: Supplementary file 1 [file jpm-13-00840-s001.zip › jpm-2381176-supplementary.pdf]

# Neuroendocrine influence on human jaw movement affected by disorders in the temporomandibular joint complex. A systematic review

## Supplementary file

### Contents

Systematic Review logic grids and search strategies ........ 2

Articles excluded upon full text review ........ 9

Cochrane GRADE critical appraisal results ........ 12

Imaging techniques applied within the included articles ........ 17

# Systematic Review logic grids and search strategies

**PubMed – Breakdown Logic Grid**

| | | |
|---|---|---|
| Mandibular movement[tiab]<br>OR<br>Symmetrical jaw movement[tiab]<br>OR<br>bilateral jaw movement[tiab]<br>OR<br>Asymmetrical jaw movement[tiab]<br>OR<br>Balanced occlusion[tiab]<br>OR<br>Joint equilibrium[tiab]<br>OR<br>Maxillomandibular motion[tiab]<br>OR<br>Jaw joint[tiab] OR<br>condyle[tiab] OR<br>mandibular growth[tiab] OR<br>Temporomandibular Joint[tiab]<br>OR<br>dental occlusion[tiab]<br>OR<br>malocclusion[tiab]<br>OR<br>Orthognathic[tiab] OR<br>Facial Asymmetry[tiab]<br><br>OR<br><br>"Mandible"[mh] OR<br>"Temporomandibular Joint"[mh]<br>OR "Dental Occlusion"[mh] OR<br>"Facial Asymmetry"[mh] | Microbio*[tiab] OR<br>Biology[tiab] OR<br>Physiology[tiab] OR<br>Hormone[tiab] OR<br>Endocrine[tiab] OR<br>Calcium[tiab] OR<br>calmodulin[tiab] OR<br>enzyme[tiab] OR<br>serotonin[tiab] OR<br>catecholamine[tiab] OR<br>estrogen[tiab] OR<br>progesterone[tiab] OR<br>testosterone[tiab] OR<br>pituitary[tiab] OR<br>cortisol[tiab] OR<br>sepsis[tiab] OR<br>cyclooxygenase[tiab] OR<br>prostaglandin[tiab] OR<br>human leukocyte antigen[tiab]<br>OR<br>transcription factor[tiab] OR<br>transforming growth factor beta[tiab] OR<br>epithelial growth factor[tiab]<br>OR<br>discoidin[tiab] OR<br>methylation[tiab]<br>OR<br>Nutrition*[tiab]<br>OR<br>Vitamin[tiab]<br>OR<br>Diabetes[tiab]<br>OR<br>Insulin[tiab]<br>OR<br>Thyro*[tiab]<br>OR<br>Iodine[tiab] | Computerised analysis[tiab]<br>OR<br>3D modelling[tiab] OR<br>Three dimensional modelling[tiab]<br>OR<br>Tomography[tiab] OR<br>Radiomic[tiab] OR<br>imaging[tiab]<br>OR<br>"tomography"[mh] |

**PubMed [Combined] - 254**

(((Mandibular movement[tiab]  OR Symmetrical jaw movement[tiab]  OR bilateral jaw movement[tiab]  OR Asymmetrical jaw movement[tiab] OR Balanced occlusion[tiab] OR Joint equilibrium[tiab] OR Maxillomandibular motion[tiab] OR Jaw joint[tiab] OR condyle[tiab] OR mandibular growth[tiab] OR Temporomandibular Joint[tiab] OR dental occlusion[tiab]  OR

malocclusion[tiab] OR Orthognathic[tiab] OR Facial Asymmetry[tiab] OR "Mandible"[mh] OR "Temporomandibular Joint"[mh] OR "Dental Occlusion"[mh] OR "Facial Asymmetry"[mh]) AND (Microbio*[tiab] OR Biology[tiab] OR Physiology[tiab] OR Hormone[tiab] OR Endocrine[tiab] OR Calcium[tiab] OR calmodulin[tiab] OR enzyme[tiab] OR serotonin[tiab] OR catecholamine[tiab] OR estrogen[tiab] OR progesterone[tiab] OR testosterone[tiab] OR thyro*[tiab] OR iodine[tiab] OR pituitary[tiab] OR cortisol[tiab] OR sepsis[tiab] OR cyclooxygenase[tiab] OR prostaglandin[tiab] OR diabetes[tiab] OR insulin[tiab] OR human leukocyte antigen[tiab] OR transcription factor[tiab] OR transforming growth factor beta[tiab] OR epithelial growth factor[tiab] OR discoidin[tiab] OR vitamin OR nutrition* OR methylation[tiab])) AND (Computerised analysis[tiab] OR 3D modelling[tiab] OR Three dimensional modelling[tiab] OR Tomography[tiab] OR Radiomic[tiab] OR imaging[tiab] OR "tomography"[mh])) NOT ("Animals"[Mh] NOT ("Animals"[Mh] AND "Humans"[Mh]))

**Scopus and WoS – Breakdown Logic Grid**

AND

- "mandibular movement" OR "symmetr* jaw movement" OR "bilateral jaw movement" OR "asymmetrical jaw movement" OR "balanced occlusion" OR "joint equilibrium" OR "maxillomandibular motion" OR "jaw joint" OR condyle OR "mandibular growth" OR "temporomandibular joint" OR "dental occlusion" OR malocclusion OR orthognathic OR "facial asymmetry"
- Hormone OR Endocrine OR Calcium OR Calmodulin OR Enzyme OR Serotonin OR Catecholamine OR Estrogen OR Progesterone OR Testosterone OR Pituitary OR Cortisol OR inflammat* OR Cyclooxygenase OR Prostaglandin OR "human leukocyte antigen" OR "chemical mediator" OR "transforming growth factor beta" OR "epithelial growth factor" OR interleukin OR discoidin
- "Computer* analysis" OR "3D model*" OR "three dimensional model*" OR tomography OR radiomic OR imaging OR "diagnostic imaging"

NOT

- Animal OR reptile OR (Animal* AND Human*)
- Knee OR “cruciate ligament” OR “hip replacement” OR spin* OR mening* OR vertebr*
- “case report” OR “case series” OR “scoping review” OR editorial OR “systematic review”
- Genetic OR gene OR epigenetic OR methylation OR “gene therap*” OR “genetic analysis”

**Scopus [Combined] – 701 results on 26.1.23**

( TITLE-ABS-KEY ( "mandibular movement" OR "symmetr* jaw movement" OR "bilateral jaw movement" OR "asymmetrical jaw movement" OR "balanced occlusion" OR "joint equilibrium" OR "maxillomandibular motion" OR "jaw joint" OR condyle OR "mandibular growth" OR "temporomandibular joint" OR "dental occlusion" OR malocclusion OR orthognathic OR "facial asymmetry" ) AND TITLE-ABS-KEY ( hormone OR endocrine OR calcium OR calmodulin OR enzyme OR serotonin OR catecholamine OR estrogen OR progesterone OR testosterone OR pituitary OR cortisol OR inflammat* OR cyclooxygenase OR prostaglandin OR "human leukocyte antigen" OR "chemical mediator" OR "transforming growth factor beta" OR "epithelial growth factor" OR interleukin OR discoidin ) AND TITLE-ABS-KEY ( "Computer* analysis" OR "3D model*" OR "three dimensional model*" OR tomography OR radiomic OR imaging OR "diagnostic imaging" ) AND NOT TITLE-ABS-KEY ( animal AND NOT reptile AND NOT ( animal* AND human* ) ) AND NOT TITLE-ABS-KEY ( knee AND NOT ( cruciate AND ligament ) AND NOT renal AND NOT ( hip AND replacement ) AND NOT spin* AND NOT mening* AND NOT vertebr* ) AND NOT TITLE-ABS-KEY ( ( case AND report ) AND NOT ( case AND series ) AND NOT ( scoping AND review ) AND NOT editorial AND NOT ( systematic AND review ) ) AND NOT TITLE-ABS-KEY ( genetic AND NOT gene AND NOT epigenetic AND NOT methylation AND NOT ( gene AND therap* ) AND NOT ( genetic AND analysis ) ) )

**Scopus [Combined] – 462 results on 27.1.23**

( TITLE-ABS-KEY ( "mandibular movement" OR "symmetr* jaw movement" OR "bilateral jaw movement" OR "asymmetrical jaw movement" OR "balanced occlusion" OR "joint equilibrium" OR "maxillomandibular motion" OR "jaw joint" OR condyle OR "mandibular growth" OR "temporomandibular joint" OR "dental occlusion" OR malocclusion OR orthognathic OR "facial

asymmetry" ) ) AND ( TITLE-ABS-KEY ( hormone OR endocrine OR calcium OR calmodulin OR enzyme OR serotonin OR catecholamine OR estrogen OR progesterone OR testosterone OR pituitary OR cortisol OR inflammat* OR cyclooxygenase OR prostaglandin OR "human leukocyte antigen" OR "chemical mediator" OR "transforming growth factor beta" OR "epithelial growth factor" OR interleukin OR discoidin ) ) AND ( TITLE-ABS-KEY ( "Computer* analysis" OR "3D model*" OR "three dimensional model*" OR tomography OR radiomic OR imaging OR "diagnostic imaging" ) ) AND NOT ( TITLE-ABS-KEY ( animal OR reptile OR ( animal* AND human* ) ) ) AND NOT ( TITLE-ABS-KEY ( knee OR "cruciate ligament" OR renal OR "hip replacement" OR spin* OR mening* OR vertebr* ) ) AND NOT ( TITLE-ABS-KEY ( "case report" OR "case series" OR "scoping review" OR editorial OR "systematic review" ) ) AND NOT ( TITLE-ABS-KEY ( genetic OR gene OR epigenetic OR methylation OR "gene therap*" OR "genetic analysis" ) )

**WOS [Combined] – 510 on 26.1.23**
(Mandibular AND movement) OR (Symmetr* AND jaw AND movement) OR (bilateral AND jaw AND movement) OR (Asymmetrical AND jaw AND movement) OR (Balanced AND occlusion) OR (Joint AND equilibrium) OR (Maxillomandibular AND motion) OR (Jaw AND joint) OR condyle OR (mandibular AND growth) OR (Temporomandibular AND Joint) OR (dental AND occlusion) OR malocclusion OR Orthognathic OR (Facial AND Asymmetry) (All Fields) and Hormone OR Endocrine OR Calcium OR Calmodulin OR Enzyme OR Serotonin OR Catecholamine OR Estrogen OR Progesterone OR Testosterone OR Pituitary OR Cortisol OR inflammat* OR Cyclooxygenase OR Prostaglandin OR (human AND leukocyte AND antigen) OR (chemical AND mediator) OR (transforming AND growth AND factor AND beta) OR (epithelial AND growth AND factor) OR interleukin OR discoidin (All Fields) and (Computerised AND analysis) OR (3D AND modelling) OR (Three AND dimensional) OR Tomography OR Radiomic OR imaging OR (diagnostic AND imaging) (All Fields) not Animal OR Reptile OR (Animal* AND Human*) (All Fields) not Knee OR (cruciate AND ligament) OR renal OR (hip AND replacement) OR spinal OR mening* OR vertebr* (All Fields) not (case AND report) OR (case AND series) OR (scoping AND review) OR editorial OR (systematic AND review) (All Fields) not Genetic OR gene OR epigenetic OR methylation OR (gene therap*) OR (genetic analysis) (All Fields)

**WOS [Combined] – 355 on 27.1.23**
"mandibular movement" OR "symmetr* jaw movement" OR "bilateral jaw movement" OR "asymmetrical jaw movement" OR "balanced occlusion" OR "joint equilibrium" OR "maxillomandibular motion" OR "jaw joint" OR condyle OR "mandibular growth" OR "temporomandibular joint" OR "dental occlusion" OR malocclusion OR orthognathic OR "facial asymmetry" (All Fields) and Hormone OR Endocrine OR Calcium OR Calmodulin OR Enzyme OR Serotonin OR Catecholamine OR Estrogen OR Progesterone OR Testosterone OR Pituitary OR Cortisol OR inflammat* OR Cyclooxygenase OR Prostaglandin OR "human leukocyte antigen" OR "chemical mediator" OR "transforming growth factor beta" OR "epithelial growth factor" OR interleukin OR discoidin (All Fields) and "Computer* analysis" OR "3D model*" OR "three dimensional model*" OR tomography OR radiomic OR imaging OR "diagnostic imaging" (All Fields) not Animal OR reptile OR (Animal* AND Human*) (All Fields) not Knee OR “cruciate ligament” OR renal OR “hip replacement” OR spin* OR mening* OR vertebr* (All Fields) not “case report” OR “case series” OR “scoping review” OR editorial OR “systematic review” (All Fields) not Genetic OR gene OR epigenetic OR methylation OR “gene therap*” OR “genetic analysis” (All Fields)

**EBSCOHost DOSS – Breakdown Logic Grid**

| | | |
|---|---|---|
| TI ("mandibular movement" OR "symmetr* jaw movement" OR "bilateral jaw movement" OR "asymmetrical jaw movement" OR "balanced occlusion" OR "joint equilibrium" OR "maxillomandibular motion" OR "jaw joint" OR condyle OR "mandibular growth" OR "temporomandibular joint" OR "dental occlusion" OR malocclusion OR orthognathic OR "facial asymmetry")<br><br>OR<br><br>AB ("mandibular movement" OR "symmetr* jaw movement" OR "bilateral jaw movement" OR "asymmetrical jaw movement" OR "balanced occlusion" OR "joint equilibrium" OR "maxillomandibular motion" OR "jaw joint" OR condyle OR "mandibular growth" OR "temporomandibular joint" OR "dental occlusion" OR malocclusion OR orthognathic OR "facial asymmetry")<br><br>OR<br><br>DE "Mandible" OR<br>DE "Temporomandibular Joint" OR DE "Dental Occlusion" | TI (Hormone OR Endocrine OR Calcium OR Calmodulin OR Enzyme OR Serotonin OR Catecholamine OR Estrogen OR Progesterone OR Testosterone OR Pituitary OR Cortisol OR inflammat* OR Cyclooxygenase OR Prostaglandin OR "human leukocyte antigen" OR "chemical mediator" OR "transforming growth factor beta" OR "epithelial growth factor" OR interleukin OR discoidin)<br><br>OR<br><br>AB (Hormone OR Endocrine OR Calcium OR Calmodulin OR Enzyme OR Serotonin OR Catecholamine OR Estrogen OR Progesterone OR Testosterone OR Pituitary OR Cortisol OR inflammat* OR Cyclooxygenase OR Prostaglandin OR "human leukocyte antigen" OR "chemical mediator" OR "transforming growth factor beta" OR "epithelial growth factor" OR interleukin OR discoidin) | TI ("Computer* analysis" OR "3D model*" OR "three dimensional model*" OR tomography OR radiomic OR imaging OR "diagnostic imaging")<br><br>OR<br><br>AB ("Computer* analysis" OR "3D model*" OR "three dimensional model*" OR tomography OR radiomic OR imaging OR "diagnostic imaging")<br><br>OR<br><br>DE "tomography" OR DE "THREE-dimensional modeling" |

**DOSS Combined - 187**

( TI ("mandibular movement" OR "symmetr* jaw movement" OR "bilateral jaw movement" OR "asymmetrical jaw movement" OR "balanced occlusion" OR "joint equilibrium" OR "maxillomandibular motion" OR "jaw joint" OR condyle OR "mandibular growth" OR "temporomandibular joint" OR "dental occlusion" OR malocclusion OR orthognathic OR "facial asymmetry") OR AB ("mandibular movement" OR "symmetr* jaw movement" OR "bilateral jaw movement" OR “sella turcica” OR "asymmetrical jaw movement" OR "balanced occlusion" OR "joint equilibrium" OR "maxillomandibular motion" OR "jaw joint" OR condyle OR "mandibular growth" OR "temporomandibular joint" OR "dental occlusion" OR malocclusion OR orthognathic OR "facial

asymmetry")  OR  DE "Mandible" OR DE "Temporomandibular Joint" OR DE "Dental Occlusion" ) AND ( TI (Hormone OR Endocrine OR Calcium OR Calmodulin OR Enzyme OR Serotonin OR Catecholamine OR Estrogen OR Progesterone OR Testosterone OR Pituitary OR Cortisol OR inflammat* OR Cyclooxygenase OR Prostaglandin OR "human leukocyte antigen" OR "chemical mediator" OR "transforming growth factor beta" OR "epithelial growth factor" OR interleukin OR discoidin)  OR  AB (Hormone OR Endocrine OR Calcium OR Calmodulin OR Enzyme OR Serotonin OR Catecholamine OR Estrogen OR Progesterone OR Testosterone OR Pituitary OR Cortisol OR inflammat* OR Cyclooxygenase OR Prostaglandin OR "human leukocyte antigen" OR "chemical mediator" OR "transforming growth factor beta" OR "epithelial growth factor" OR interleukin OR discoidin) ) AND ( TI ("Computer* analysis" OR "3D model*" OR "three dimensional model*" OR tomography OR radiomic OR imaging OR "diagnostic imaging")  OR  AB ("Computer* analysis" OR "3D model*" OR "three dimensional model*" OR tomography OR radiomic OR imaging OR "diagnostic imaging")  OR  DE "tomography" OR DE "THREE-dimensional modeling" )

**Embase – Logic grid breakdown (343)**

<table>
<tr>
<td rowspan="2">(Mandibular movement<br>OR<br>Symmetrical jaw movement<br>OR<br>bilateral jaw movement<br>OR<br>Asymmetrical jaw movement<br>OR<br>Balanced occlusion<br>OR<br>Joint equilibrium<br>OR<br>Maxillomandibular motion OR<br>Jaw joint OR<br>condyle OR<br>mandibular growth OR<br>Temporomandibular Joint OR<br>dental occlusion<br>OR<br>malocclusion<br>OR<br>Orthognathic OR<br>Facial Asymmetry).ti,ab<br><br>OR<br><br>Exp Mandible OR<br>Exp Temporomandibular Joint<br>OR exp tooth occlusion OR<br>face asymmetry.sh</td>
<td rowspan="2">(Microbio* OR<br>Biology OR<br>Physiology OR<br>Hormone OR<br>Endocrine OR<br>Calcium OR<br>calmodulin OR<br>enzyme OR<br>serotonin OR<br>catecholamine OR<br>estrogen OR<br>progesterone OR<br>testosterone OR<br>pituitary OR<br>cortisol OR<br>sepsis OR<br>cyclooxygenase OR<br>prostaglandin OR<br>human leukocyte antigen OR<br>transcription factor OR<br>transforming growth factor<br>beta OR<br>epithelial growth factor OR<br>discoidin OR<br>methylation).ti,ab</td>
<td>(Computer* analysis OR<br>3D modelling OR<br>Three dimensional<br>modelling<br>OR<br>Tomography OR<br>Radiomic OR<br>Imaging).ti,ab<br><br>OR<br><br>Tomography.sh OR exp<br>three-dimensional imaging</td>
</tr>
<tr>
<td>NOT: (exp animal/ or animal<br>experiment/ or nonhuman/)<br>not (exp human/ or<br>human experiment/)</td>
</tr>
</table>

## Articles excluded upon full text review

| Author | Article title | Reason for exclusion |
|---|---|---|
| Soo Min, 2020 | Local Injection of Growth Hormone for Temporomandibular Joint Osteoarthritis | Animal study |
| Hamid, 2020 | Dose-Dependent Clinical, Radiographic, and Histopathologic Changes of 17β-Estradiol Levels Within the Temporomandibular Joint: An Experimental Study | Animal study |
| Jiao, 2018 | Experimental study on mandibular length and facial symmetry of low estrogen level and anterior disc displacement of temporomandibular joint | Animal study |
| Deniz, 2013 | Analysis of hormone relaxin in the synovial fluid of patients with temporomandibular disorders | Conference proceeding |
| Han, 2010 | Influence of intra-articular corticosteroid on the efficacy of arthrocentesis in treatment of temporomandibular joint anterior disc displacement without reduction | Full English text not found |
| Nemtoi, 2013 | Quantitative and qualitative bone assessment of the posterior mandible inpatients with diabetes mellitus: a cone beam computed tomography study | Full English text not found |
| Taner, 2019 | Volumetric and three-dimensional examination of sella turcica by cone-beam computed tomography: reference data for guidance to pathologic pituitary morphology | Full English text not found |
| Averyanov, 2021 | Systemic approach to diagnosis and treatment planning of occlusion anomalies in adult patients with dentition defects and deformities residing in the iodine-deficient region | Full English text not found |
| Tanghiloo, 2020 | Relationship between the dimensions of Sella Turcica with the vertical skeletal pattern of the face on cone beam computed tomography (CBCT) images in an Iranian population | Full English text not found |

| | | |
|---|---|---|
| Mikami, 2014 | Cytopathologic Diagnosis on Joint Lavage Fluid for Patients with Temporomandibular Joint Disorders | No neuroendocrine component documented |
| Matsumoto, 2006 | Cytokine profile in synovial fluid from patients with internal derangement of the temporomandibular joint: A preliminary study | No neuroendocrine component documented |
| Nakawaki, 2017 | Growth hormone receptor gene variant and three-dimensional mandibular morphology | No neuroendocrine component documented |
| Murakami, 1998 | Intra-articular levels of prostaglandin E2, hyaluronic acid, andchondroitin-4 and -6 sulfates in the temporomandibular joint synovialfluid of patients with internal derangement | No neuroendocrine component documented |
| Bartosz, 2020 | Association of Estrogen Receptor 1 and Tumor Necrosis Factor αPolymorphisms with Temporomandibular Joint Anterior Disc Displacement without Reduction | No neuroendocrine component documented |
| Cevidanes, 2014 | 3D osteoarthritic changes in TMJ condylar morphology correlates with specific systemic and local biomarkers of disease | No neuroendocrine component documented |
| Bollhalder, 2020 | Magnetic resonance imaging followup of temporomandibular joint inflammation, deformation, and mandibular growth in juvenile idiopathic arthritis patients receiving systemic treatment | No neuroendocrine component documented |
| Voog, 2004 | Progression of radiographic changes in the temporomandibular joints of patients with rheumatoid arthritis in relation to inflammatory markers and mediators in the blood | No neuroendocrine component documented |
| Dasgupta, 2017 | Sella Turcica Bridging As A Predictor Of Class II Malocclusion–An Investigative Study | No neuroendocrine component documented |
| Voog, 2003 | Inflammatory mediators and radiographic changes in temporomandibular joints of patients with rheumatoid arthritis | No neuroendocrine component documented |

| | | |
|---|---|---|
| Ulmner, 2020 | Synovial tissue cytokine profile in disc displacement of the temporomandibular joint | No radiographic references of occlusion or TMJ provided |
| Rodic, 2021 | Bone quality analysis of jaw bones in individuals with type 2 diabetesmellitus-post mortem anatomical and microstructural evaluation | No radiographic references of occlusion or TMJ provided |
| Ito, 2021 | Computed tomography texture analysis of mandibular condylar bone marrow indiabetes mellitus patients | No radiographic references of occlusion or TMJ provided |
| Almalki, 2022 | Association of Salivary IGF and IGF/IGFBP-3 Molar Ratio with Cervical Vertebral Maturation Stages from Pre-Adolescent to Post-Adolescent Transition Period—A Cross-Sectional Exploratory Study | No radiographic references of occlusion or TMJ provided |
| Habibi, 2012 | Safety and efficacy of US-guided CS injection into temporomandibular joints in children with active JIA | No radiographic references of occlusion or TMJ provided |
| Jolly, 2013 | Assessment of Maxillary and Mandibular Bone Density in Controlled Type IIDiabetes: A Computed Tomography Study | No radiographic references of occlusion or TMJ provided |
| Sumukh, 2021 | The Comparative Evaluation of the Morphology and Dimensions of the Sella Turcica in Skeletal Class III Patients and Patients With Unilateral Cleft Lip and Palate in Post-Pubertal Age Group | No radiographic references of occlusion or TMJ provided |
| Hezam, 2020 | Computed Tomography Evaluation of Sella Turcica Dimension in Skeletal Class III Malocclusion Among Adult Ukrainian Peoples | No radiographic references of occlusion or TMJ provided |
| Doetzer, 2021 | Association of estrogen receptor alpha 1 and TMJ dysfunction: A pilot study | No radiographic references of occlusion or TMJ provided |
| Hirahara, 2022 | Quantitative assessment of the mandibular condyle in patients with diabetes mellitus using difusion-weighted magnetic resonance imaging | No radiographic references of dynamic parameters of occlusion or TMJ provided |

# Cochrane GRADE critical appraisal results

| Certainty assessment | | | | | | | № of patients | | Effect | | Certainty | Importance |
|---|---|---|---|---|---|---|---|---|---|---|---|---|
| № of studies | Study design | Risk of bias | Inconsistency | Indirectness | Imprecision | Other considerations | TMJ disorders | Control | Relative (95% CI) | Absolute (95% CI) | | |
| **Antonarakis, 2018** | | | | | | | | | | | | |
| 1 | observational studies | serious[a,b] | not serious | not serious | not serious | all plausible residual confounding would reduce the demonstrated effect | 0 cases 0 controls | | not estimable | - | ⊕⊕◯◯ Low | |
| | | | | | | | - | 0.0% | | | | |
| **Arabshahi, 2005** | | | | | | | | | | | | |
| 1 | observational studies | serious[c,d,e,f] | not serious | not serious | not serious | all plausible residual confounding would reduce the demonstrated effect | | | not estimable | | ⊕⊕◯◯ Low | |
| **Fred, 2020** | | | | | | | | | | | | |
| 1 | observational studies | not serious | not serious[g,h] | not serious | not serious | all plausible residual confounding would suggest spurious effect, while no effect was observed | | 0.0% | not estimable | | ⊕⊕⊕◯ Moderate | |
| **Lochbuhler, 2015** | | | | | | | | | | | | |
| 1 | observational studies | not serious | not serious | not serious | not serious | all plausible residual confounding would reduce the demonstrated effect dose response gradient | | | not estimable | | ⊕⊕⊕⊕ High | |
| **Moystad, 2007** | | | | | | | | | | | | |
| 1 | randomised trials | not serious[i] | not serious | serious[j] | not serious | all plausible residual confounding would reduce the demonstrated effect dose response gradient | | | not estimable | | ⊕⊕⊕⊕ High | |
| **Ringold, 2008** | | | | | | | | | | | | |

| Certainty assessment | | | | | | | № of patients | | Effect | | Certainty | Importance |
|---|---|---|---|---|---|---|---|---|---|---|---|---|
| № of studies | Study design | Risk of bias | Inconsistency | Indirectness | Imprecision | Other considerations | TMJ disorders | Control | Relative (95% CI) | Absolute (95% CI) | | |
| 1 | observational studies | not serious | serious[h,k] | not serious | not serious | all plausible residual confounding would reduce the demonstrated effect<br>dose response gradient | | | not estimable | | ⨁⨁⨁◯<br>Moderate | |
| **Stoll, 2012** | | | | | | | | | | | | |
| 1 | observational studies | not serious[l] | not serious | not serious | not serious | all plausible residual confounding would reduce the demonstrated effect | | | not estimable | | ⨁⨁⨁◯<br>Moderate | |
| **Yavuz, 2018** | | | | | | | | | | | | |
| 1 | randomised trials | not serious | not serious | serious[m,n] | not serious | none | | | not estimable | | ⨁⨁⨁◯<br>Moderate | |
| **Berg, 2009** | | | | | | | | | | | | |
| 1 | observational studies | not serious | not serious | not serious | not serious | all plausible residual confounding would reduce the demonstrated effect<br>dose response gradient | | | not estimable | | ⨁⨁⨁⨁<br>High | |
| **Richey, 1995** | | | | | | | | | | | | |
| 1 | observational studies | not serious | not serious | serious[o] | not serious | strong association<br>all plausible residual confounding would reduce the demonstrated effect | 0 cases 0 controls<br>- | 0.0% | not estimable | - | ⨁⨁⨁◯<br>Moderate | |
| **Masoud, 2012** | | | | | | | | | | | | |
| 1 | observational studies | serious[p] | not serious | not serious | not serious | all plausible residual confounding would suggest spurious effect, while no effect was observed<br>dose response gradient | | | not estimable | | ⨁⨁⨁◯<br>Moderate | |
| **Gus, 2015** | | | | | | | | | | | | |

| Certainty assessment | | | | | | | № of patients | | Effect | | Certainty | Importance |
|---|---|---|---|---|---|---|---|---|---|---|---|---|
| № of studies | Study design | Risk of bias | Inconsistency | Indirectness | Imprecision | Other considerations | TMJ disorders | Control | Relative (95% CI) | Absolute (95% CI) | | |
| 1 | observational studies | serious[q,r] | serious[s] | not serious | not serious | strong association | | | not estimable | | ⊕◯◯◯ Very low | |
| **Hajati, 2009** | | | | | | | | | | | | |
| 1 | observational studies | not serious | not serious | not serious | not serious | all plausible residual confounding would reduce the demonstrated effect dose response gradient | | | not estimable | | ⊕⊕⊕⊕ High | |
| **Shen, 2022** | | | | | | | | | | | | |
| 1 | observational studies | serious[t] | not serious | not serious | not serious | all plausible residual confounding would reduce the demonstrated effect | | | not estimable | | ⊕⊕◯◯ Low | |
| **Suenaga, 2001** | | | | | | | | | | | | |
| 1 | observational studies | not serious | not serious | not serious | not serious | all plausible residual confounding would reduce the demonstrated effect | | | not estimable | | ⊕⊕⊕◯ Moderate | |
| **Xiong, 2019** | | | | | | | | | | | | |
| 1 | observational studies | serious[u] | not serious | not serious | not serious | all plausible residual confounding would suggest spurious effect, while no effect was observed dose response gradient | | | not estimable | | ⊕⊕⊕◯ Moderate | |
| **Leszczyszyn, 2021** | | | | | | | | | | | | |
| 1 | observational studies | serious[v,w] | not serious | not serious | serious[x] | none | | | not estimable | | ⊕◯◯◯ Very low | |
| **Feldreich, 2012** | | | | | | | | | | | | |
| 1 | | serious[y] | serious[z] | not serious | not serious | | 0 cases 0 controls | | | - | | |

| Certainty assessment | | | | | | | № of patients | | Effect | | Certainty | Importance |
|---|---|---|---|---|---|---|---|---|---|---|---|---|
| № of studies | Study design | Risk of bias | Inconsistency | Indirectness | Imprecision | Other considerations | TMJ disorders | Control | Relative (95% CI) | Absolute (95% CI) | | |
| | observational studies | | | | | all plausible residual confounding would reduce the demonstrated effect dose response gradient | - | 0.0% | not estimable | | ⊕⊕◯◯ Low | |
| **Kajii, 2005** | | | | | | | | | | | | |
| 1 | observational studies | serious[aa] | not serious | not serious | not serious | none | | | not estimable | | ⊕◯◯◯ Very low | |

**CI:** confidence interval

## Explanations

a. patients who underwent corticosteroid therapy had smaller mouth openings at baseline comparison

b. Case and control groups were not equally distributed

c. Different compositions of exogenous corticosteroids applied.

d. Dosage applied based on empirical evaluation

e. 82% of the patients received NSAIDs at some point of the treatment

f. Not all participants underwent MRI imaging

g. Corticosteroid class was switched from Methylprednisolone to Triamcinolone

h. Details of patients receiving the two forms of exogenous corticosteroid were not mentioned

i. Relied on a subjective scoring system to compare signs of bilateral involvement

j. Dosage of exogenous administration were not mentioned

k. Imaging modality was neither standardised, nor adequately documented across all patients

l. The lack of randomisation and control group could influence the selection and interpretation of the reports

m. Events during follow-up requiring re-administration of corticosteroid was not documented

n. Scoring of imaging scans were not documented

o. Details from radiographic evaluation were not mentioned

p. Orientation of neck stature during cervical spine radiograph can distort the intervertebral space and lead to inappropriate classifications

q. No healthy control was established

r. No quantitative approaches followed for the measurement of occlusal parameters

s. Possibilities of causation and correlation were not reported

t. Methods for recording jaw movement parameters were not documented

u. Age range of control group not adequately matched

v. Biases involving radiographic interpretation were not described

w. Classes were not equally distributed

x. The outcomes surrounding the provision of D3 supplements hinges on patient truthfulness

y. Baseline mouth opening values for healthy control was not provided.

z. Conditions and outcomes for patients receiving different imaging scans were not documented

aa. Jaw movement measurements were not recruited from healthy volunteers as an eligibility criterion

## Imaging techniques applied within the included articles

| **Author, year** | **Type of radiomic data used** |
|---|---|
| Antonarakis, 2018 | MRI (1.5T Avanto, Siemens; Germany) with Gadolinium contrast enhancement<br><br>T1 sequence<br>• TR:TE = 576:13<br>• Slice = 2.5mm<br><br>T2 sequence<br>• TR:TE = 3330:94<br>• Slice = 3mm<br><br>Proton density<br>• TR:TE = 2580:21<br>• Slice = 3mm<br>• |
| Arabshahi, 2005 | MRI with Gadolinium contrast enhancement. (Scanning parameters were not mentioned) |
| Frid, 2020 | MRI scan [(3T Magnetom Skyra, Siemens; Germany) or (1.5T Magnetom Aera or Avanto, Siemens; Germany)]<br><br>T1 Sequence<br>• TR:TE = 681:8 avg<br>• Slice = 2mm<br><br>T2 Sequence<br>• TR:TE = 3500:71 avg<br>• Slice = 2mm<br><br>Proton Density<br>• TR:TE = 3470:32 avg<br>Slice = 2mm |
| Lochbuhler, 2015 | MRI Scan (1.5T Signa, GE Medical Systems; USA) with gadolinium contrast enhancement<br><br>T1 Sequence<br>• TR:TE = 600:11<br>• Slice = 2mm<br><br>T2 Sequence<br>• TR:TE = 2840:86<br>• Slice = 2mm<br><br>Proton Density<br>• TR:TE = 2660:25<br>• Slice = 2mm |
| Moystad, 2007 | CT scan (Lightspeed Ultra 3x, GE Medical Systems, USA)<br><br>• V:A = 120kV:80mA |

| | |
|---|---|
| | FOV = 16 x 16 cm |
| Ringold, 2008 | CT scans and MRI (Details were not mentioned) |
| Stoll, 2012 | MRI<br><br>T1 sequence<br>• TR:TE = 600:14ms<br>• Slice = 2-3mm<br><br>T2 Sequence<br>• TR:TE = 3500:105ms<br>• Slice = 2-3mm<br><br>Proton density<br>• TR:TE = 3500:15ms<br>Slice = 2-3mm |
| Yauvz, 2018 | MRI scan (details were not mentioned) |
| Berg, 2009 a | MRI scan (Magnetom Sonata; Siemens Medical Systems, Germany) with gadopentetate dimeglumine oral contrast<br>• Amplitude = 40 mT/m<br>• Slew rate = 200 mT/m per ms<br><br>Lateral cephalograpms taken in centric occlusion at 1.5m distance from X-ray source |
| Richey, 1995 b | 2D radiographs. Details were not mentioned |
| Masoud, 2012 c | Lateral cephalograms and cervical radiographs. Details were not mentioned |
| Gus, 2015 | MRI (Vantage Atlas-X; Toshiba, Japan) |
| Hajati, 2009 | CT scan (NewTom DVT mod 9000; Verona, Italy) |
| Shen, 2022 | MRI scan (Ingenia; Philips Healthcare Systems, Netherlands)<br><br>Proton Density<br>• TR:TE = 2000”20 ms<br>• FOV = 11cm<br>Slice = 1mm (0.3mm skip) |
| Suenaga, 2001 | MRI scan (Signa; GE Medical Systems, USA)<br><br>T1 sequence<br>• TR:TE = 635:30ms (300:12 for fat-suppression)<br>• Slice = 3mm<br>• FOV = 13cm<br><br>T2 sequence<br>• TR:TE = 3000:100ms<br>Slice = 5mm |
| Xiong, 2019 | CBCT (details not mentioned) |
| Leszczyszyn, 2021 | 2D radiography (Intraoral, Panoramic and cephalometric radiography) |
| Feldreich, 2012 | CT and MRI scans. (Detailed description not provided) |
| Kajii, 2005 | MRI scan (details were not provided) |

MRI, Magnetic Resonance Imaging; TR:TE, repetition time: echo time (in ms); CT, Computed Tomography; V:A, ratio of voltage and current applied; FOV, Field of View (in cm)
